# Blocking of the CXCR4-CXCL12 Interaction Inhibits the Migration of Chicken B Cells Into the Bursa of Fabricius

**DOI:** 10.3389/fimmu.2019.03057

**Published:** 2020-01-10

**Authors:** Maria Laparidou, Antonina Schlickenrieder, Theresa Thoma, Kamila Lengyel, Benjamin Schusser

**Affiliations:** ^1^Reproductive Biotechnology, School of Life Sciences Weihenstephan, Technical University of Munich, Freising, Germany; ^2^Department of Behavioural Neurobiology, Max-Planck-Institut for Ornithology, Seewiesen, Germany

**Keywords:** B lymphocytes, CXCR4, CXCL12, chicken, bursa, migration, SDF-1, B cell receptor

## Abstract

B cells have first been described in chickens as antibody producing cells and were named after the Bursa of Fabricius, a unique organ supporting their development. Understanding different factors mediating the early migration of B cells into the bursa of Fabricius is crucial for the study of B cell biology. While CXCL12 (stromal derived factor 1) was found to play an important role in B lymphocyte trafficking in mammals, its role in the chicken is still unknown. Previous studies indicated that chicken CXCL12 and its receptor CXCR4 are simultaneously expressed during bursal development. In this study, we investigated whether the CXCR4/CXCL12 interaction mediates B cell migration in chicken embryo. We used the CRISPR/Cas9 system to induce a CXCR4 knockout in chicken B cells which led to chemotaxis inhibition toward CXCL12. This was confirmed by adoptive cell transfer and inhibition of the CXCR4/CXCL12 interaction by blocking with the small inhibitor AMD3100. In addition, we found that the chicken exhibits similarities to mice when it comes to CXCR4 being dependent on B cell receptor expression. B cells lacking the B cell receptor failed to migrate toward CXCL12 and showed no response upon CXCL12 stimulation. Overall, we demonstrated the significance of CXCR4/CXCL12 in chicken B cell development *in vivo* and the importance of the B cell receptor in CXCR4 dependent signaling.

## Introduction

B cells were first recognized in the bursa of Fabricius as antibody producing cells (1). Despite the progress made in the field of B cell biology, there is still need for better understanding of various aspects in B cell development. Originating from hematopoietic cells in the dorsal aorta (2, 3), B cell precursors are first located in the embryonic spleen (4). However, in order to further develop, they need to reach the bursa. Therefore, around embryonic day 10 they migrate with the blood stream (5, 6) toward the bursa of Fabricius where they organize themselves in follicles (7) and undergo diversification and proliferation (8, 9). Through several rounds of gene conversion the chicken's antibody repertoire is generated (10). Finally, around hatch B cells emigrate from the bursa to be found later in secondary peripheral lymphoid tissues (11). Though the developmental journey of the B cells is known, the driving mechanisms of this pathway are still unclear. It has been shown that although the successful rearrangement and expression of the surface immunoglobulin is essential for the emigration of cells out of the bursa (12), it is not essential for the B cells to migrate into the bursa (12, 13).

In mammals the migration of the B cell precursors toward the bone marrow, the site of proliferation and maturation equivalent to the avian bursa, is driven by chemokine signaling. Specifically, the migration is highly regulated by the interaction of the CXCR4 chemokine receptor with its ligand CXCL12, also known as stroma derived factor-1 (SDF-1). CXCR4 is expressed by the B cells while CXCL12 is expressed by the bone marrow stroma cells (14). The same chemokine receptor and ligand have been shown to be key players in many other migration processes, including the mobilization of mammalian hematopoietic stem cells, lymphocytes (14–16) but also primordial germ cells (17). CXCR4 and its ligand CXCL12 are both expressed in chickens during B cell development (18–21). It has been suggested that immature B cells might also migrate toward the bursa under the influence of the CXCR4/CXCL12 interaction. However, this was not proven so far.

Therefore, the goal of the current study was to investigate the role of CXCL12 mediated signaling via CXCR4 for the migration of B cells toward the bursa of Fabricius. In this study, we were able to inhibit chemokinesis toward the chemokine CXCL12, by generating a CXCR4 knockout chicken B cell line with the CRISPR/Cas9 system. *In vivo* experiments using AMD3100 to block the interaction of CXCR4 with CXCL12 highlighted their significance for the migration of B cells toward the bursa. Since in mice the function of the CXCR4 receptor is dependent on the B cell receptor (BCR) expression (22), we investigated B cell receptor knockout chicken B cells (BCR^neg^) in chemotaxis assays to examine if this also applies in the chicken. BCR^neg^ B cells failed to migrate toward the chemokine CXCL12. Furthermore, CXCL12 stimulation did not result in calcium signaling as seen in the case of wt B cells. This study demonstrates the significance of CXCR4 and CXCL12 in chicken B cell development *in vitro* and *in vivo*, but also the importance of the B cell receptor expression for CXCR4 dependent signaling.

## Materials and Methods

### Animals

Fertilized chicken eggs from white leghorn chickens and immunoglobulin heavy chain knockout (JH-KO) chicken were provided from the animal facility of the Chair of Reproductive Biotechnology, at the Technical University of Munich (School of Life Sciences, Weihenstephan, Freising, Germany), where white leghorn chickens were kept and provided with free access to water and a standard chicken diet *ad libitum*. Eggs were incubated at 37.8°C temperature, 55% humidity and rocking three times per day. Eggs were opened and the embryos were euthanized by decapitation. All experiments were performed in accordance with the German Animal Protection Act.

### Isolation of Embryonic Splenocytes

Embryonic spleens were prepared and directly set in 1 ml of cold PBS. Depending on the age of the donor embryos, the splenocytes were prepared either individually or in pools of two to five spleens. Spleens were homogenized by resuspending them in the PBS with a 1 ml syringe. Cell suspensions were centrifuged at 400 g for 4 min. The supernatant was discarded and cells were counted for further processing.

### Leukocyte Isolation From the Embryonic Bursa

Depending on the experiment requirements bursae were prepared either separately or in pools after euthanizing the embryos. In case of pool preparation, the bursae were prepared and set on ice until further process. Bursae were passed through a 100 μm cell strainer by means of a 1 ml syringe plunger and 2 ml PBS. The cell suspension was collected into a petri dish. In order to isolate the leukocytes, a density gradient centrifugation was performed with Biocoll separating solution (Biochrom, Berlin, Germany) according to the manufactures instructions.

### Adoptive Cell Transfer

For the adoptive cell transfer, incubated eggs were prepared as described before (23). Embryonic splenocytes were isolated from the donor embryos as described in 2.2. Per host embryo 1 × 10^6^ cells were transferred, diluted in 50 μl PBS. Prior to the injection of the cells into the embryo, cells were filtered through a cell strainer (50 μm pores). Cells were prepared fresh for three to four embryos each time.

### Genotyping

Blood sampling was performed from embryos between ED8 and ED16 as described before. Genotyping was performed as described by Schusser et al. (12).

### Blocking of the CXCR4 Chemokine Receptor on the Cell Surface

In order to block the CXCR4 receptor on the surface of the cells (either splenocytes or DT40 cells), cells were incubated with the CXCR4 antagonist, AMD3100 (Sigma Aldrich, Taufkirchen, Germany) (200 μg/ml in PBS) for 15 min at room temperature.

### FACS Analysis

Cell suspensions of 1 × 10^6^ cells each were plated and stained with following primary antibodies: anti-chCXCR4, clone 9D9 (0.07 μg/ml) (Bio-Rad, Feldkirchen, Germany), anti-chBu1, clone AV20 (0.625 μg/ml) (Southern Biotech, Alabama, United States), anti-chIgM, clone M1 (2.5 μg/ml) (Southern Biotech, Alabama, United States) or the anti-ch Bu1-FITC (2.5 μg/ml) (Southern Biotech, Alabama, United States). For the detection of the primary unconjugated antibodies the following secondary antibodies were used: anti-muIgG (H+L)-APC (5 μg/ml), anti-muIgG1-AlexaFluor 647 (1.25 μg/ml). Both secondary antibodies were produced by Southern Biotech, Alabama, United States. Cells were filtered through a 100 μm mesh to remove cell clumps and transferred into FACS tubes. Live/dead staining was performed with the 7-AAD Viability Staining Solution (5 μg/ml) (BioLegend, San Diego, California, United States) shortly before flow cytometry. FACS analysis was performed with the BD Accuri C6 (BD Biosciences). As controls unstained cells as well as secondary antibody only controls were included in all analyses. As positive controls wt cells were included in the analyses. Analysis of the data was performed with FlowJo10 v10 (BD, Franklin Lakes, New Jersey, United States).

### Knock Out of CXCR4 in DT40 Cells

A CRISPR/Cas9 plasmid was generated by modification of px330 (Addgene #42230), inserting a guide RNA (gRNA) targeting CXCR4 and the gene for hygromycin B resistance. Design of the specific gRNA was carried out with the Online Tool CRISPR Design (http://crispr.mit.edu). GRNA oligos (5′-CACCGgatttgctgacaatggctcgg-3′, 5′-AAACccgagccattgtcagcaaatcC-3′) were synthesized by Eurofins Genomics (Ebersberg, Germany). Cloning was performed as described before (24). DT40 cells were cultured in Dulbecco's Modified Eagle's medium (Biochrom, Berlin, Germany) supplemented with 10% fetal bovine serum (Biochrom, Berlin, Germany), 1% chicken serum (Life Technologies, Darmstadt, Germany), 1% Glutamax (Life Technologies, Darmstadt, Germany), and 0.2% 2-Mercaptoethanol (Life Technologies, Darmstadt, Germany). Cells were maintained at 37°C, 5% CO_2_. DT40 cell electroporation was performed with Cell Line Nucleofector Kit V (Lonza, Cologne, Germany) according the manufacturing protocol and BTX™ Harvard Apparatus ECM™ 830 Electroporation Generator (350 V, 125 μs/pulse, 8 pulses). A total of 10 μg DNA of the CRISPR/Cas9 vector were used per electroporation. At 24 h post-electroporation, the cells were treated with hygromycin B (3,000 μg/ml) (PanReac Applichem, Darmstadt, Germany) for 48 h. After hygromycin B selection, limiting dilution was performed to achieve single cell clones. Single cell clones were further expanded and tested by flow cytometry for absence of the CXCR4. For the analysis, the cells were stained as described before.

Genomic DNA from DT40 cells was isolated as described by Wang et al. (25). To analyse CRISPR/Cas9 gene editing, the target locus was amplified using FIREPol® MasterMix (Solis BioDyne, Tartu, Estonia) according to manufactures instructions using specific primers: forward 5′-CTTACTCCTCAGAGAAACACAAAGG-3′ and reverse 5′-TGACATGAACTGCCTTACACAGG-3′ (Eurofins Genomics, Ebersberg, Germany) and sequenced at Eurofins Genomics. Sequence analysis was carried out using SeqmanPro (DNAStar, Madison, USA).

### Immunofluorescence Histology

Immunofluorescence histology was performed as described by Schusser et al. (12). B cells were detected with an anti-chBu1, Clone AV20-FITC antibody (2.5 μg/ml) (Southern Biotech, Alabama, United States). The BCR was detected using anti-chIgM, clone M1 antibody (1.25 μg/ml) (Southern Biotech, Alabama, United States) followed by anti-muIgG-AlexaFluor 568 antibody (10 μg/ml) (Fisher Scientific, Schwerte, Germany). Stainings of the sections with secondary antibody only were included in the analysis. Sections of wt embryos bursae were included as positive control in the analysis. Microscopy and documentation were performed using an AxioSkop, AxioCam MRc5 system, and AxioVision software (Zeiss, Jena, Germany).

### Chemotaxis Assay

Chemotaxis assay were performed as described before (20). Briefly transwell plates (Sigma-Aldrich, Taufkirchen, Germany) were coated with 50 μl fibronectin (10 μg/ml) and then incubated for 1 h at 37° and 5% CO_2_. Fibronectin was removed and the plates were set to dry at 37° for 2 h. Cells were counted and washed with RPMI medium twice. Five million cells of each sample were diluted in chemotaxis medium (48.75 ml RPMI 1640 with Glutamax with 1.25 ml 20% endotoxin-free BSA) without SDF-1 (eBioscience, California, United States). A 500 μl volume of the chemokine dilutions or the chemotaxis medium without chemokine were plated in the bottom chamber. The cell suspension was plated in the upper chamber or in the case of the 100% positive control sample, directly in the bottom chamber. The probes were set for incubation for one and a half hour (37°C and 5% CO_2_). The bottom chamber was washed and cells were then resuspended. 500 μl of the suspension was diluted in 400 μl Fluo-buffer (5 g Bovine Serum Albumin—Fraction V9, 50 mg Sodium azide add 500 ml PBS pH 7.2, all by AppliChem, Darmstadt, Germany). By absolute counting the migrated cells were measured and statistical analysis was performed. As chemokinesis control, 500 μl of chemotaxis medium with SDF-1 (eBioscience, California, United States) at a 100 ng/ml concentration were set in the bottom chamber while 100 μl were set in the upper chamber. Assay was always done in triplicates. Throughout the process, the cells were not exposed to temperatures under 21°.

### Calcium Mobilization Assay

A 50 μg aliquot of Fluo-4 AM (FisherScientific, Schwerte, Germany) were diluted in 50 μl dimethyl sulfoxide (DMSO) to create a solution of 1 μg/μl. After counting the cells, one million cells were washed in calcium and magnesium free PBS (CMF-PBS) with 2% chicken serum and 1 g/l glucose. Cells were resuspended in 1 ml of the above medium. A 0.5 μl volume of Fluo-4 (at a 0.5 μM final concentration) were added to each sample and incubated for 20 min at room temperature. Cells were washed three times with CMF-PBS medium and then resuspended in 0.5 ml of the medium for analysis. The control probe was measured for 30 s, then 50 μl of anti-chIgM (10 μg/ml) or anti-CXCL12 (SDF-1) (100 ng/ml) (eBioscience, California, United States) were added to the probes and analysis were continued for another 300 s.

### Analysis of the CXCR4 and CXCL12 Expression in the Embryonic Bursa

Bursae were set in lysis tubes (InnuSpeed tubes, type A, Analytik Jena, Jena, Germany) and were homogenized (SpeedMill, Analytik Jena, Jena, Germany) according to the protocol of the ReliaPrep™ RNATissue Miniprep System (Promega, Mannheim, Germany). RNA was isolated from homogenized tissue according to the manufacturer instructions. The concentration of the isolated RNA was defined by NanoDrop Photometer measurement and the quality of the RNA was tested with the Agilent RNA 6000 Nano Kit according to the manufacturer protocol using a 2100Bioanalyzer (Agilent, California, United States). cDNA was synthesized according to manufactures instructions using GoScript Reverse Transcription System (Promega, Mannheim, Germany) and stored at −20°C. The qRT-PCR reaction and the cycling parameters were set and performed according to manufacturer's protocol using GoTaq® qPCR Master Mix (Promega, Mannheim, Germany). The primers used for the qRT-PCR were adapted from Busalt et al. (26) and are found in Supplemental Table 1.

## Results

### Migration of B Lymphocytes in the Blood During Embryonic Development

**Figure 1:**
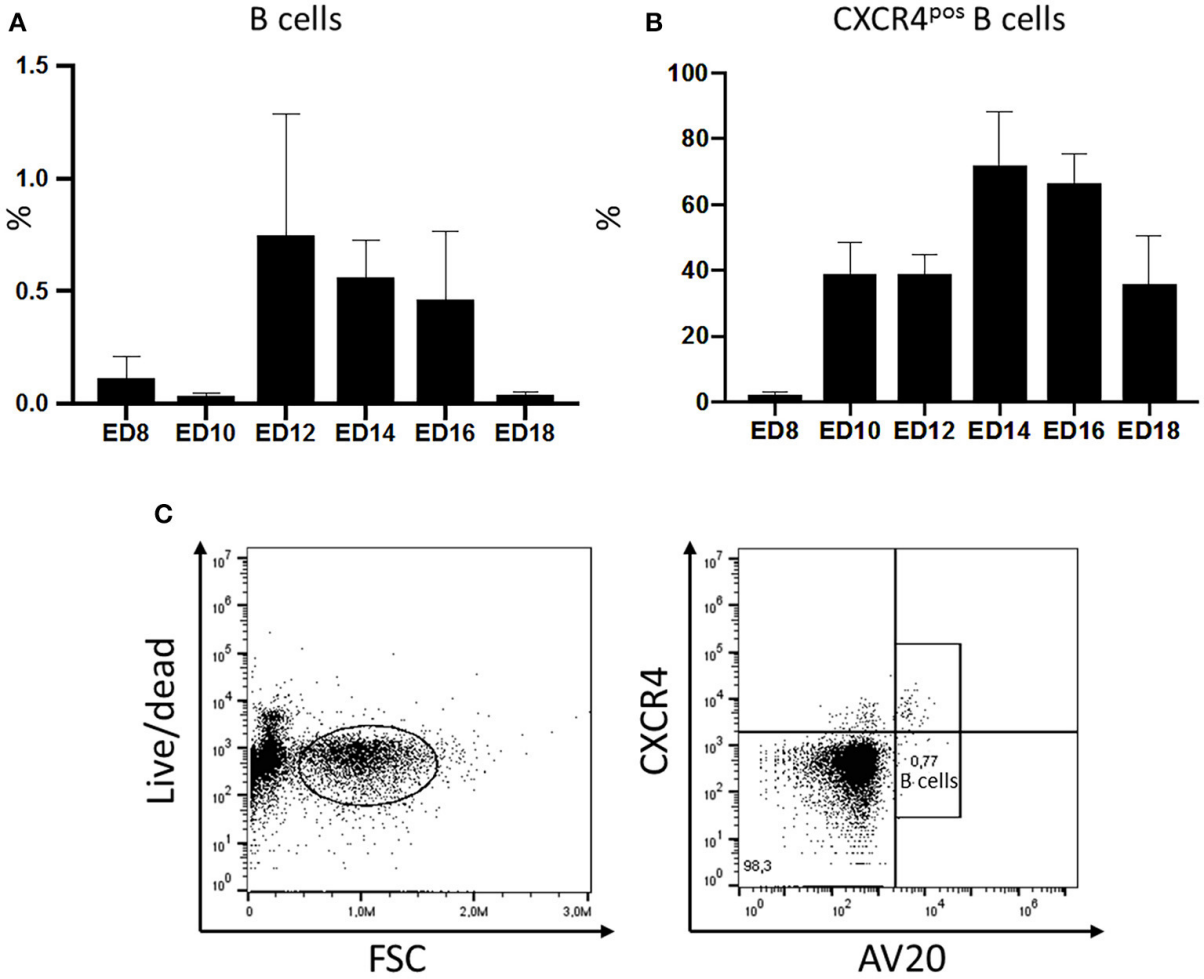
Migration of chicken B cells during embryonic development and CXCR4 expression on B cells detected in the embryonic blood between ED8 and ED18. After blood sampling a density gradient centrifugation was performed. **(A)** The isolated cells were stained with the B cell marker AV20 for FACS analysis (*n* ≥ 3, not normal distributed per Kolmogorov-Smirnov and Shapiro-Wilk tests, non-parametric analysis, Kruskal-Wallis, *^=^*p* < 0.05). **(B)** The amount of CXCR4^pos^ B cells was examined by double staining with the B cell marker AV20 and the anti-chCXCR4 antibody between ED8 and ED18. Live cells were gated and the CXCR4 expression of the AV20^pos^ B cells **(C)** was evaluated (*n* ≥ 3, data normally distributed per Kolmogorov-Smirnov and Shapiro-Wilk tests, independent *t*-test analysis, *p* < 0.05).

The migration of B cells with the blood was analyzed during embryonic development by flow cytometry. Blood was taken from embryos on ED8 and on every other day till ED18 and leukocytes were isolated and stained for B lymphocytes. The relative amounts of B cells were examined and compared. The first B cells were already detected on ED8 even though in very low levels (0.127%). On the following days, migrating cells increased to reach a peak on ED12 with 0.7% of the peripheral blood mononuclear cells (PBMC). Toward hatch, the relative amount of B cell decreased to 0.04% (Figure 1A).

### Migrating B Cells Express CXCR4 on Their Surface

*In ovo* blood sampling (Supplemental Figure 1) followed by FACS analysis enabled a close examination of the migrating B cells. It was possible to control if B cells migrating with the blood already express the CXCR4 receptor. Therefore, PBMCs were isolated and double stained with the chicken B cell marker AV20 and an antibody against chicken CXCR4 (Figure 1C). On ED8 2.38% of the B cells were already expressing the CXCR4 chemokine receptor on their surface. On ED10 the percentage of B cells expressing the receptor rose to 38.96% and remained till ED12 at the same levels. On ED14 there was a rapid increase of CXCR4^pos^ B cells to 72% of the B cell population. Toward hatch the percentage started to decrease again, down to 35.9% on ED18 (Figure 1B).

### Knock Out as Well as Chemical Blocking of the CXCR4 Chemokine Receptor Prevent Chemotaxis

**Figure 2:**
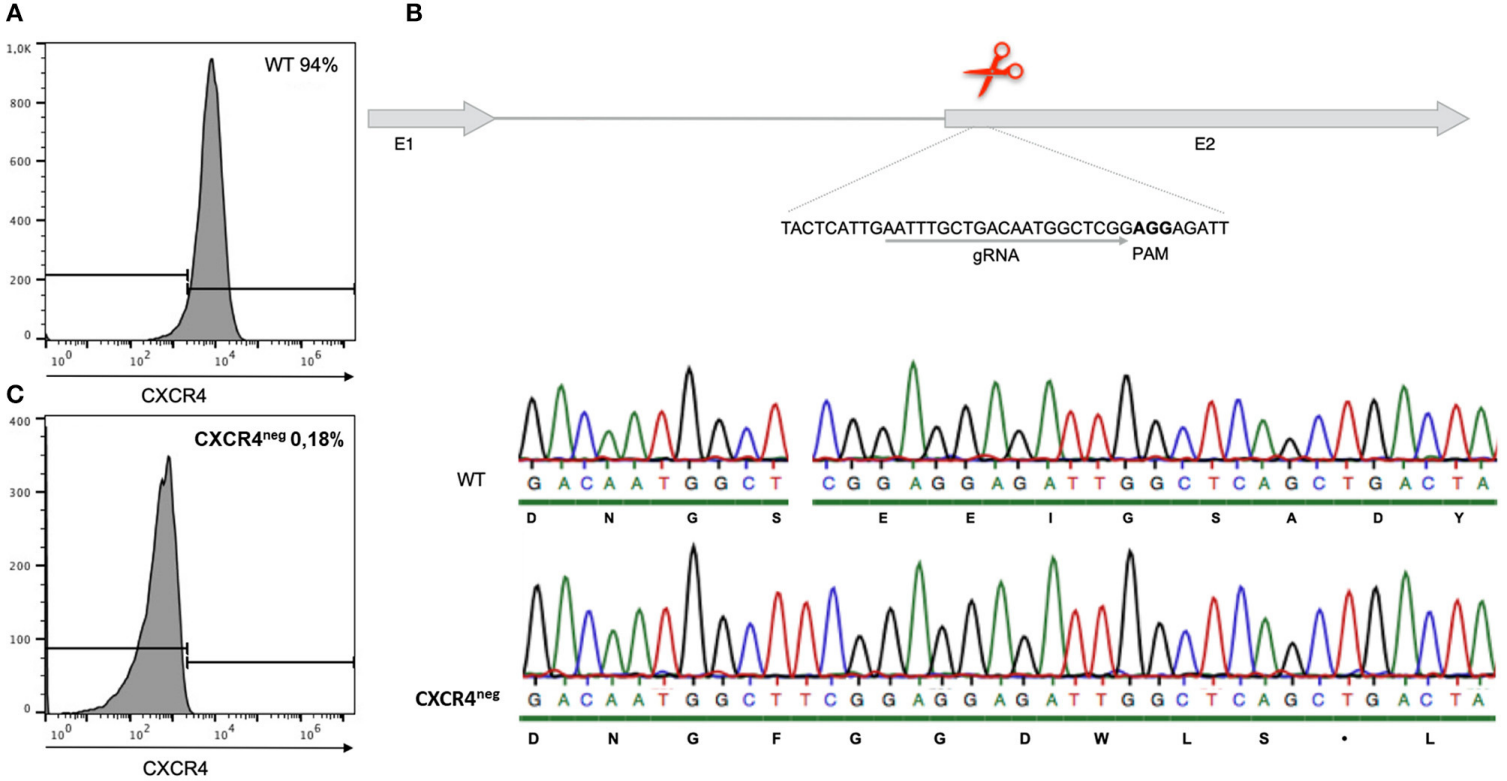
Gene editing of CXCR4 with the CRISPR/Cas9 system in chicken DT40 cells. **(A)** CXCR4 gene structure with guide RNA (gRNA) recognition site and protospacer-adjacent motif (PAM) sequence. **(B)** Sequence analysis of CXCR4^neg^ and wt DT40 cells with amino acid sequence. CXCR4^neg^ cell sequence analysis revealed a T insertion causing a frameshift and therefore generation of a premature stop codon. **(C)** Flow cytometry analysis of CXCR4^neg^ and wt cells with staining for CXCR4. Gene editing successfully knocked out the CXCR4 chemokine receptor.

Cells of the chicken B cell line DT40 were checked by staining with a chicken specific anti-CXCR4 antibody for chemokine receptor expression by flow cytometry. Ninety-five percent of the cells confirmed to be positive for CXCR4 (Figure 2A). Chemotaxis assays using CXCL12 showed migration of DT40 cells toward the ligand. However, in order to evaluate the significance of the CXCR4/CXCL12 mediated signal, the assay was repeated with blocking or knockout of the CXCR4 receptor.

**Figure 3:**
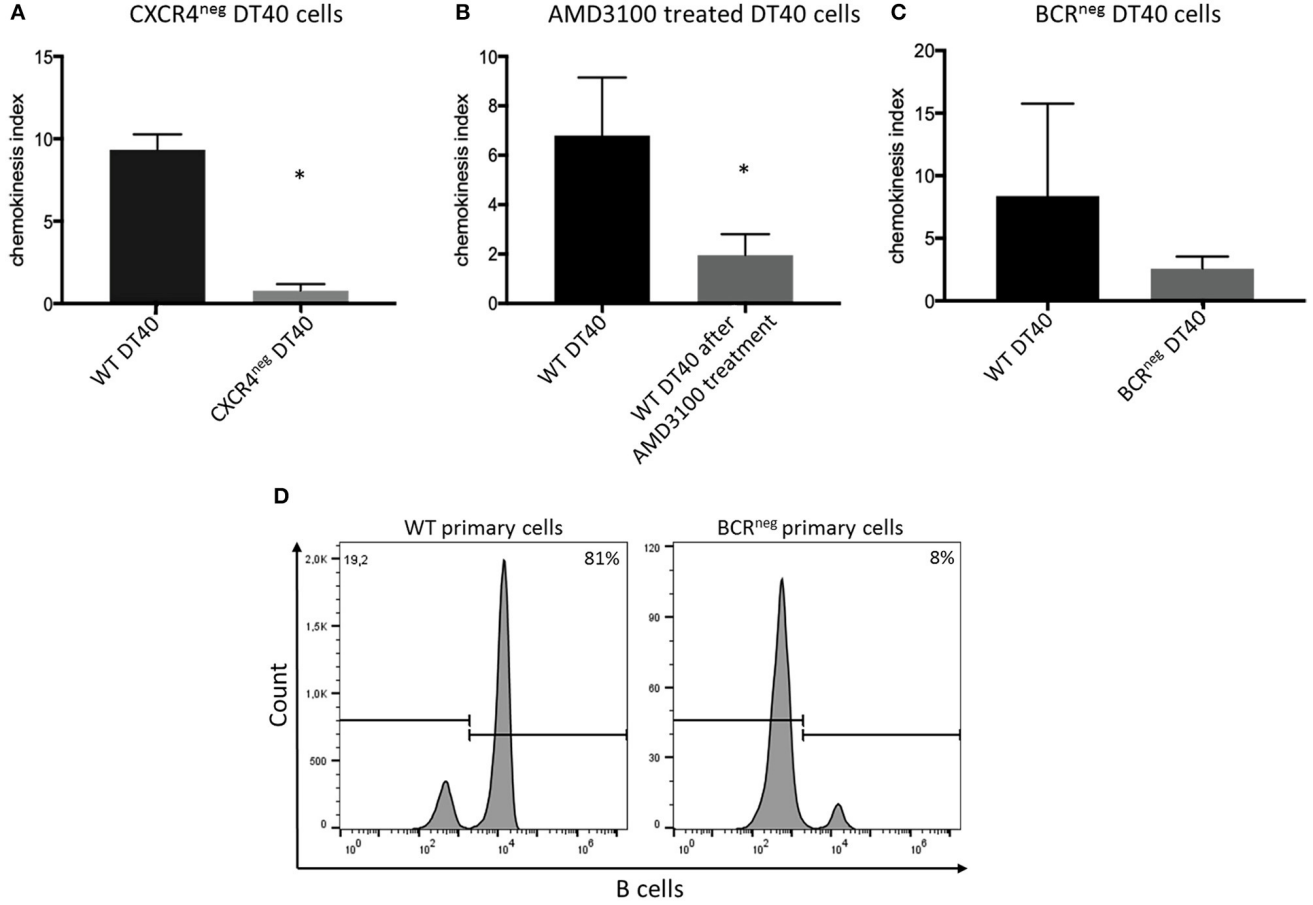
CXCR4/CXCL12 dependent migration of DT40 cells and primary bursal cells. The DT40 cell migration seen in chemotaxis assays was inhibited by **(A)** knocking out the CXCR4 receptor in the DT40 cell line **(A)**, Treating the cells with the CXCR4 antagonist, AMD3100 also led to cell migration inhibition **(B)**. Finally, DT40 cells lacking the B cell receptor migrated in much lower levels toward the chemokine **(C)** CXCL12 in all cases *n* ≥ 3, data normally distributed per Kolmogorov-Smirnov and Shapiro-Wilk tests, independent *t*-test analysis, *p < 0.05 in case of **(A,B)** and *p* > 0.05 in case of **(C)**. In addition chemotaxis assays using primary bursal B cells were performed as described before. The migration of the BCR^neg^ cells toward CXCL12 was compromised as seen in **(D)**. While 81% of the wt cells migrated toward CXCL12 only 8% of the BCR^neg^ cells migrated toward CXCL12. One representative FACS analysis is shown.

The CXCR4 receptor was inhibited by chemical blocking, achieved by treating the cells with AMD3100. DT40 cells treated with AMD3100 did not migrate toward the chemokine ligand (Figure 3).

However, in an attempt to completely exclude the effect of the receptor-ligand interaction, CXCR4 was successfully knocked out by gene editing (Figure 2B). Through CRISPR/Cas9 gene editing a DT40 cell line showing no expression of the CXCR4 receptor was generated (Figure 2C). The CXCR4^neg^ DT40 cells, in contrast to CXCR4^pos^ DT40 cells, did not migrate toward the chemokine CXCL12 (Figure 3).

Both chemical and genetic inhibition of the receptor led to inhibition of the cell migration.

### CXCR4 Induced Ca^2+^ Signaling Is Dependent on Surface B Cell Receptor Expression

Previously generated B cell receptor (BCR) knockout DT40 cells (27), completely lacking the BCR were analyzed for their ability to migrate toward CXCL12. The BCR^neg^ DT40 cells failed to migrate toward the chemokine confirming that CXCR4/CXCL12 induced chemotaxis is dependent on surface expression of the BCR in chicken B lymphocytes (Figure 3).

Moreover, in order to investigate if primary cells also exhibit the same behavior, primary bursal B cells were isolated from BCR^neg^ embryos and used in migration assays. Primary B cells lacking the BCR receptor, though expressing CXCR4, also failed to migrate toward the chemokine CXCL12. FACS analysis after chemotaxis assay is shown (Figure 3).

**Figure 4:**
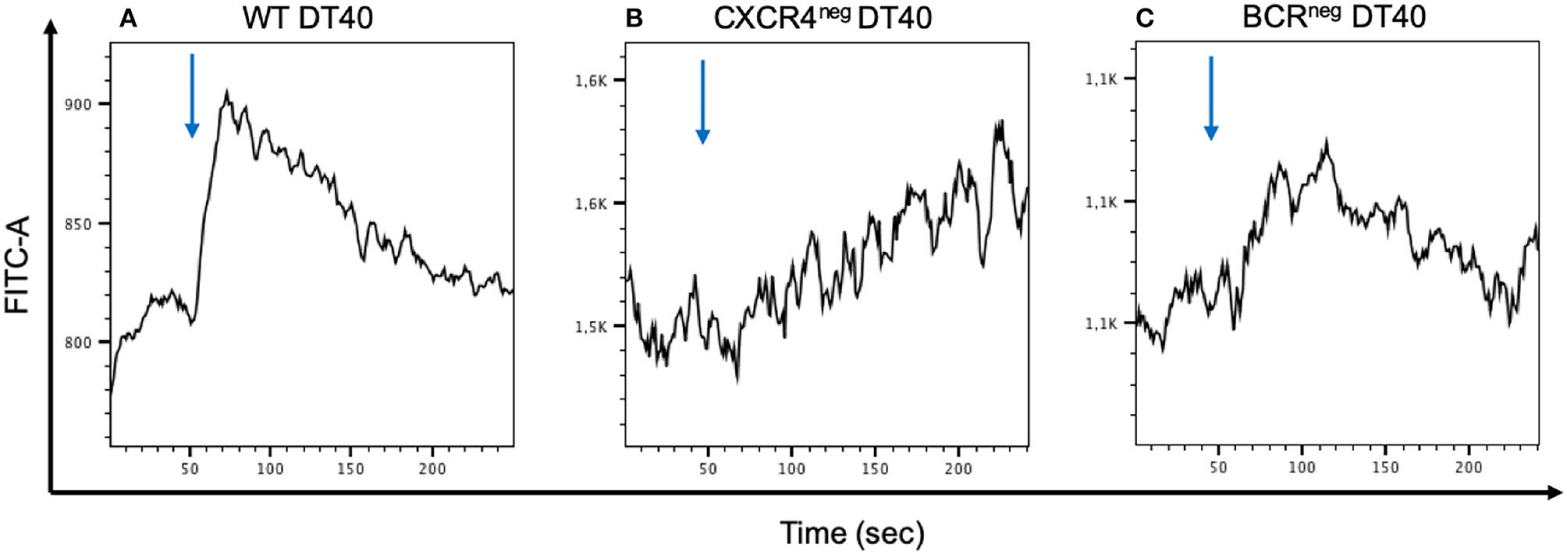
Calcium mobilization signaling through CXCR4/CXCL12 interaction is inhibited when the CXCR4 receptor or the BCR receptor is inhibited. WT, CXCR4^neg^, and BCR^neg^ DT40 cells have been labeled with FLUO-4 and stimulated with CXCL12 in order to detect calcium release by flow cytometry. Signal activity occurring from the CXCR4/CXCL12 interaction in wt cells **(A)** is interrupted in CXCR4^neg^ DT40 cells **(B)**. A similar effect is observed in BCR^neg^ DT40 cells **(C)**.

To examine the CXCR4 Ca^2+^ signaling capacity CXCR4^pos^, CXCR4^neg^, and BCR^neg^ DT40 cells were stimulated with CXCL12 and the calcium flux was measured by flow cytometry. The assay demonstrated that the signaling seen in the CXCR4^pos^ cells was clearly hampered in both CXCR4^neg^ and BCR^neg^ DT40 cells (Figure 4).

### *In vivo* Inhibition of the CXCR4/CXCL12 Interaction by AMD3100 Results in Inhibition of the B Cell Migration Into the Bursa of Fabricius

**Figure 5:**
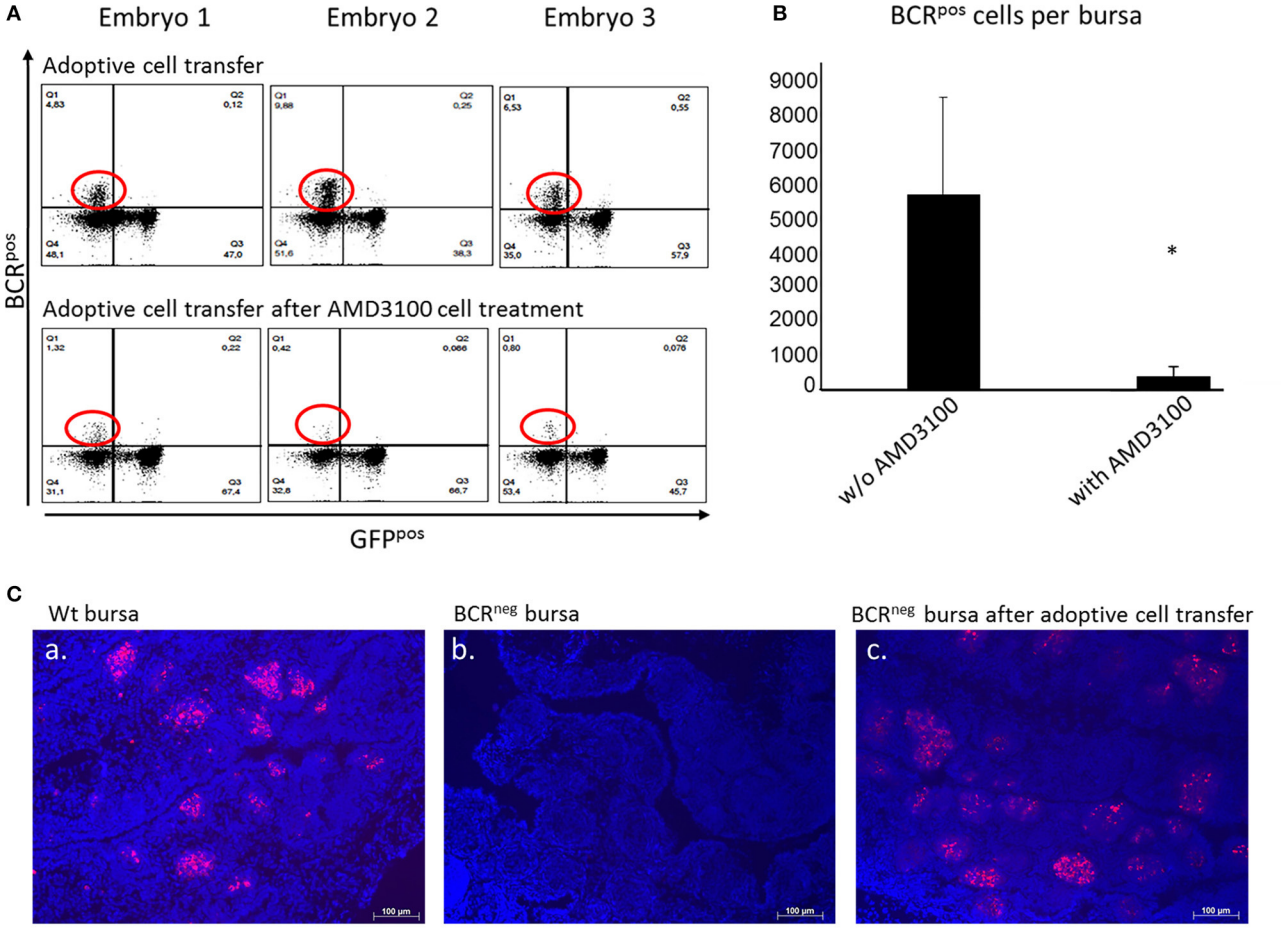
Adoptive cell transfer experiment with and without AMD3100 treatment of the transferred cells. **(A)** Bursae of the embryos were tested for BCR^pos^, GFP^neg^ cells after wt cell transfer into BCR^neg^ embryos on ED19. The detection was performed by staining with the anti-chicken IgM antibody. In the bursae of BCR^neg^ embryos in which wt splenocytes were injected (**A**, top panel), up to 17% BCR^pos^/GFP^neg^ cells were detected (**A**, top panel, population circled in red). In the recipients of wt cells after AMD3100 treatment this population was limited to 1% (**A**, bottom panel, population circled in red). **(B)** After detection of wt IgM^pos^ cells in the bursae of the recipient BCR^neg^ embryos per flow cytometry and absolute counting, the amount of the transferred cells detected in the bursa were compared. In the bursae of the embryos, in which AMD3100 treated cells (*n* = 3) were transferred were significantly less IgM^pos^ cells detected than in the bursae of the embryos in which splenocytes were transferred without AMD3100 treatment (*n* = 6) (normally distributed per Shapiro-Wilk and Kolmogorov-Smirnov tests, independent *t*-test, *p < 0.05). **(C)** Fluorescence histology staining of the nuclei with DAPI (in blue) and the B cell receptor with the antiIgM antibody (in red) of a wt embryonic bursa (a), of a BCR^neg^ embryonic bursa (b) and of a BCR^neg^ embryonic bursa after adoptive cell transfer of wt splenocytes (c). The presence of BCR^pos^ B cells is clearly demonstrated in the BCR^neg^ embryonic bursa. B cells are organized in bursal follicles as seen in the wt bursa. Representative results out of at least three examined animals per group are shown.

To also confirm the importance of CXCR4 on B cell migration *in vivo*, ED14 splenocytes were isolated from BCR^pos^ embryos and transferred into host BCR^neg^ embryos of the same age. On ED19, the embryos were euthanized and their bursae were examined by FACS analysis. BCR^pos^ cells, originating from the wild type donor, were detected in the bursae of BCR^neg^ embryos into which BCR^pos^ splenocytes were transferred (Figure 5A, top panel). Thereby, it was shown that BCR^pos^ donor cells have the ability to migrate toward the bursa, colonize the organ and form follicles. At least 2% of the total bursal B cell population of the BCR^neg^ recipient embryo was BCR^pos^. Immunhistochemistry confirmed the formation of B cell follicles after adoptive transfer of pre-bursal stem cells (Figure 5C).

After establishing the adoptive transfer and showing that B cells maintain their capacity to colonize the bursa in a host embryo, further experiments were performed. The purpose was to investigate if blocking of the CXCR4 receptor would result in inhibition of the cell migration *in vivo*, thereby interfering with the ability of the donor cells to migrate toward the bursa where CXCL12 is widely expressed. Once again, WT embryos were used as donors and BCR^neg^ embryos as host. Splenocytes from the WT embryos were isolated on ED14 and prepared for cell transfer into the homozygous BCR^neg^ embryos. The BCR^neg^ host embryos were separated in two groups, one control group in which the cells were transferred and one group in which the cells were transferred after they were treated with AMD3100. The bursae were prepared on ED19 and stained for the BCR and analyzed by flow cytometry (Figure 5A). By absolute counting, the BCR^pos^/GFP^neg^ cells were estimated per bursa and statistical analyses were performed. The green fluorescent protein expression (GFP) of the cells was taken into consideration since BCR^neg^ embryos are GFP^pos^ and the green fluorescence can be a further parameter for the control of the origin of the cells. In the mock treated embryos, the mean number of BCR^pos^ cells detected in the bursa was 5,692 donor BCR^pos^ cells while in the embryos which received cells treated with AMD3100, only 378 cells made it on average to the host's bursa. The blocking of CXCR4 reveals a significant reduction of the immigrating cells (Figure 5B).

### Embryos Lacking the B Cell Receptor Exhibit Higher CXCL12 Expression in Their Bursa

**Figure 6:**
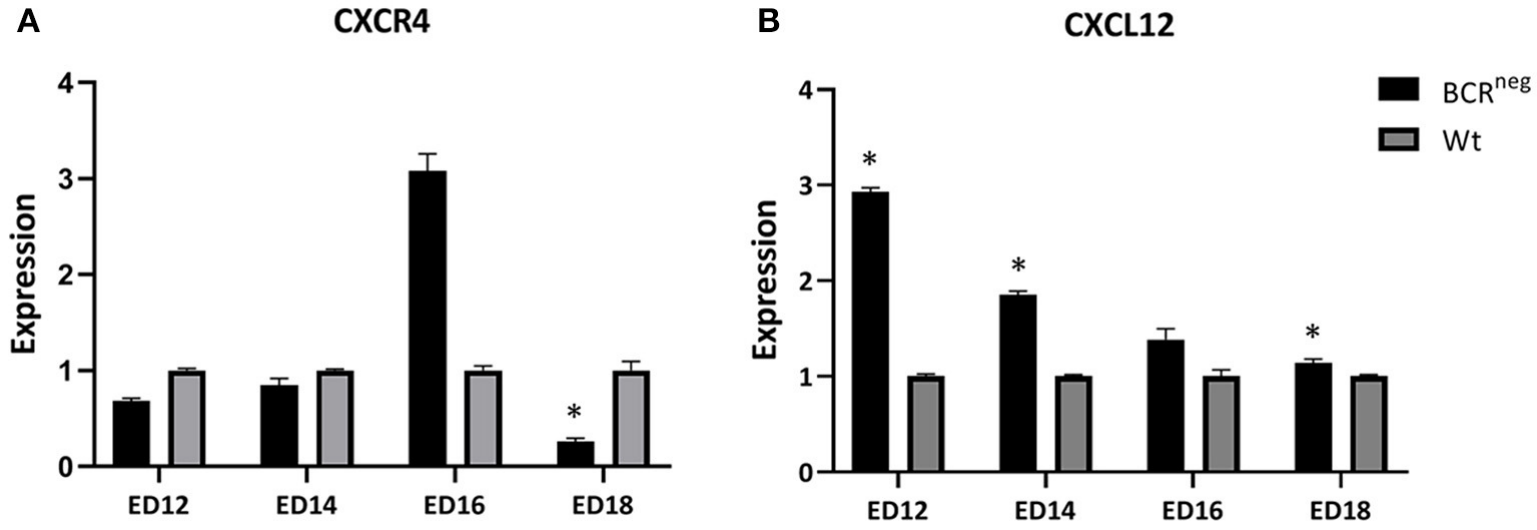
Expression of CXCR4 and CXCL12 in the embryonic bursa of BCR^neg^ in comparison to the wt embryonic bursa from ED12 to ED18. Bursae of BCR^neg^ and wt embryos were prepared every second day from ED12 and on, till ED18. The RNA was isolated and cDNA was generated to perform qPCR in order to examine the levels of expression in the BCR^neg^ embryos only in comparison to the wt embryos. The diagram is not representing the expression in wt embryos throughout the time, but the BCR^neg^ in comparison to the wt on every time point. Expression levels were calculated using the ddCT method and is represented as 2^−ddCT^. Error bars show the SEM dCT values (ED12 and ED14: Wt *n* = 4 and BCR^neg^ *n* = 3, ED16: WT and BCR^neg^ *n* = 3, ED18: Wt *n* = 3, and BCR^neg^ *n* = 5. Data is in all cases normally distributed per Shapiro-Wilk and Kolmogorov-Smirnov tests. *Depicts significance in the dCT values difference per Levene test, *p* < 0.05).

The expression of CXCR4 and CXCL12 were examined in the bursa of Fabricius of wild type and BCR^neg^ embryos during development. Bursae of the above-mentioned embryos were prepared on ED12, ED14, ED16, and ED18 and RNA was isolated and expression analyses were performed. Interestingly, the BCR^neg^ embryos exhibited higher CXCL12 expression than the same aged wt embryos (Figure 6). The highest difference between wt and BCR^neg^ embryos expression was seen on ED12, at the peak of the B cell migration. The expression of CXCL12 was three times higher in the embryos lacking the B cell receptor in comparison to the wt embryos.

## Discussion

Though the bursa of Fabricius has been recognized since decades as the organ of maturation, diversification and proliferation of the developing B lymphocytes (28, 29), the regulating signals for their migration toward the bursa have still not been clarified. Previous studies have shown that the expression of the B cell receptor on the cell surface is not essential for the cell migration (12, 30).

B lymphocyte precursors are known to migrate with the bloodstream and into the bursal mesenchyme between embryonic day 8 and 15 (31, 32). This knowledge has been gained by blood smears and histology (4, 31). *In ovo* blood sampling enabled the closer examination of the B cell migration patterns with the bloodstream during embryonic development. It was possible to detect the first B cells even though in minimal numbers already on ED8. The B cells numbers increased in the blood stream toward ED12 which is thereby assumed to be the peak of the migration period. Until ED16, there are high percentages of circulating B lymphocytes, while on ED18, when the migration of the cells has long been completed (33), only a minimal amount of B cells can be detected. Our analysis confirms that B cell migration takes place between ED8 and ED18 and is setting the peak of the migration on ED12.

We analyzed the expression of the CXCR4 chemokine receptor on the surface of the migrating B cells. The CXCR4 presence has been previously analyzed in the embryonic spleen and bursa (18, 21). An increase of the CXCR4 expression was observed in the bursa during the colonization period (26). This was thought to be due to the rising numbers of CXCR4 expressing B cells which arrive in the bursa. *In ovo* blood sampling enabled also the analysis of the CXCR4 expression by the B cells throughout embryonic life. As expected CXCR4 is expressed on the B cell surface at the onset of the B cell migration indicating the possible significance of the CXCR4 signaling in the migration of the cells.

Chemotaxis assays showed that CXCR4 expressing B cells are migrating toward CXCL12. To investigate if migration toward CXCL12 happens in a CXCR4-dependent manner we used reverse genetics and small inhibitors. We first blocked CXCR4 by chemical inhibition with AMD3100 and then by knocking out of the CXCR4 receptor with the CRISPR/Cas9 system in the chicken B cell line DT40. Both methods led to almost complete inhibition of the cell migration demonstrating that the migration is CXCR4/CXCL12 dependent.

Since all *in vitro* experiments consistently showed that B cell migration is driven by the CXCR4/CXCL12 interaction, further experiments were performed *in vivo*. We established adoptive cell transfers to first examine if donor B cells maintain their ability to migrate toward the bursa of Fabricius and second to examine if donor B cells with blocked CXCR4 receptor can migrate toward the CXCL12 expressing host bursa. After standardizing the adoptive cell transfer in the chicken embryo, we were able to track the donor cells in the recipient embryos bursa. We demonstrated that transplanted B cells are able to colonize the host bursa. However, when the donor lymphocytes were treated with the CXCR4 antagonist, AMD3100, the migration of the transferred cells was inhibited.

It has been clearly demonstrated that CXCL12 is a chemoattractant for CXCR4 expressing cells (34). However, we wanted to examine if this happens in an autonomous way. Therefore, we investigated the role of surface BCR in CXCL12 mediated Ca^2+^ signaling via the CXCR4 receptor. We stimulated two genetically modified chicken B cell lines in comparison to unmodified cells with CXCL12. Cells lacking either CXCR4 or BCR did not respond to CXCL12 stimulation compared to wt cells, revealing the necessity of the BCR receptor for CXCL12 mediated calcium signaling. This finding engages results from previous studies in mice which have supported that the CXCR4/CXCL12 dependent migration of B lymphocytes requires the presence of the B cell receptor on the cell surface. The same studies suggest that CD19 is probably the signal mediator of the CXCR4/CXCL12 interaction with the assistance of the BCR (22). This seems very plausible since this would not be the only case where CD19 is essential for signal transduction induced by the BCR (35). In the chicken, even though there are hints for the existence of CD19, so far there are no information about CD19 expression and its role in chicken B cells available.

In addition to calcium signaling experiments, we further investigated the role of the BCR in the CXCR4/CXCL12 driven migration. Therefore, we used BCR^neg^ primary cells as well as BCR^neg^ DT40 B cells (12, 27). Interestingly, both BCR^neg^ primary and BCR^neg^ DT40 cells, failed to migrate toward CXCL12 even though B cells in BCR^neg^ animals are known to be migrating into the bursa of Fabricius (12). Since demonstrating that surface BCR expression is needed for CXCR4/CXCL12 mediated B cell migration, we assume that *in vivo* CXCR4/CXCL12 is not driving the immigration of B lymphocytes into the bursa of Fabricius exclusively and that other factors can also navigate B cell migration in chickens.

Finally, we examined if the absence of the B cell receptor in the BCR^neg^ embryos results in differences in the expression of the CXCR4 and CXCL12 levels. Therefore, we compared the levels of CXCL12 expression in the embryonic bursa of the BCR^neg^ embryos to the expression levels found in the wt embryonic bursa. BCR^neg^ embryos, expressed higher levels of CXCL12 in their bursa between ED12 and ED18 (Figure 6). The highest difference though, was seen on ED12 at the peak of the B lymphocyte migration period. Since we demonstrated that the B cell receptor is essential for the CXCR4/CXCL12 driven migration and since BCR^neg^ cells lack the BCR receptor, we consider the possibility of the stromal cells upregulating their CXCL12 expression in an attempt to trigger the recruitment of more B cells. However, this is a theory that would require further experiments to be confirmed.

Furthermore, on ED16 there is a higher expression of CXCR4 in the BCR^neg^ embryonic bursa in comparison to the wt bursa. Previous findings support that there are two different types of CXCR4^pos^ populations found in the bursa, one expressing CXCR4 in high levels (CXCR4^pos high^) and the other one expressing low levels of CXCR4 (CXCR4^pos low^) (26). Interestingly, CXCR4^pos high^ B cells are also OV^neg^. The OV antigen, also known as chicken L-12 antigen, is being expressed only by cells able and ready to emigrate from the bursa (36). On the other hand, B cells that are OV^neg^ have been shown to be incapable of emigration. Consequently, since B cells in the BCR^neg^ embryos are incapable of emigrating out of the bursa, the finding of high CXCR4 expression in their bursa is consistent with the theory that CXCR4^pos high^ are OV^neg^ and don't emigrate from the bursa.

Overall, we demonstrated that the CXCR4/CXCL12 signaling mediates B cell migration in the chicken. However, CXCR4/CXCL12 driven B cell migration is not independent and further requires the expression of the B cell receptor by the B cell in order to trigger the initial signaling.

## Data Availability Statement

The datasets generated for this study are available on request to the corresponding author.

## Ethics Statement

All experiments were performed in accordance with the German Animal Protection Act.

## Author Contributions

ML, AS, KL, and TT performed the experiments. ML and BS planned the experiment and analyzed the data. ML, KL, and BS wrote the manuscript.

### Conflict of Interest

The authors declare that the research was conducted in the absence of any commercial or financial relationships that could be construed as a potential conflict of interest.

## Supplementary Material

Supplemental Figure 1 *In ovo* blood sampling and adoptive cell transfer. **(A)** Eggs from a BCR^pos/neg^ × BCR^pos/neg^ breeding were set for incubation. On ED11 blood was taken *in ovo* for genomic DNA isolation and genotyping of the embryos in order to detect the BCR^neg^ embryos. On ED14 splenocytes isolated from wt embryos were adoptively transferred into the BCR^neg^ embryos. On ED18 the embryos were sacrificed and bursal lymphocyte populations were analyzed per flow cytometry. *In ovo* blood sampling but also adoptive cell transfer require drilling of a triangle over a stable vessel **(B)**, dispatching of the triangle from the egg shell **(C)**, viewing of the vessel after putting a drop of paraffin oil on the membrane **(D)**, and approach of the vessel with a 30G needle **(E)**.

Supplemental Table 1 Primers used for the qRT-PCR for the analysis of the CXCR4 and CXCL12 expression in the embryonic bursa.

## References

[1] Glick B The bursa of fabricus and antibody production. Poult Sci. (1956) 35:224–5. 10.3382/ps.0350224

[2] Dieterlen-Lievre F. On the origin of haemopoietic stem cells in the avian embryo: an experimental approach. J Embryol Exp Morphol. (1975) 33:607–19. 1176862

[3] Lassila O Eskola J Toivanen P Dieterlen-Lievre F. Lymphoid stem cells in the intraembryonic mesenchyme of the chicken. Scand J Immunol. (1980) 11:445–8. 10.1111/j.1365-3083.1980.tb00011.x 6966820

[4] Houssaint E Mansikka A Vainio O. Early separation of B and T lymphocyte precursors in chick embryo. J Exp Med. (1991) 174:397–406. 10.1084/jem.174.2.397 1856628 PMC2118911

[5] Weber WT Foglia LM. Evidence for the presence of precursor B cells in normal and in hormonally bursectomized chick embryos. Cell Immunol. (1980) 52:84–94. 10.1016/0008-8749(80)90402-5 6993016

[6] Houssaint E Torano A Ivanyi J. Ontogenic restriction of colonization of the bursa of Fabricius. Eur J Immunol. (1983) 13:590–5. 10.1002/eji.1830130715 6135615

[7] Olah I Glick B. The number and size of the follicular epithelium (FE) and follicles in the bursa of Fabricius. Poult Sci. (1978) 57:1445–50. 10.3382/ps.0571445 724605

[8] Reynaud CA Anquez V Grimal H Weill JC. A hyperconversion mechanism generates the chicken light chain preimmune repertoire. Cell. (1987) 48:379–88. 10.1016/0092-8674(87)90189-9 3100050

[9] Thompson CB Neiman PE. Somatic diversification of the chicken immunoglobulin light chain gene is limited to the rearranged variable gene segment. Cell. (1987) 48:369–78. 10.1016/0092-8674(87)90188-7 3100049

[10] Arakawa H Kuma K Yasuda M Ekino S Shimizu A Yamagishi H. Effect of environmental antigens on the Ig diversification and the selection of productive V-J joints in the bursa. J Immunol. (2002) 169:818–28. 10.4049/jimmunol.169.2.818 12097385

[11] Paramithiotis E Ratcliffe MJ. B cell emigration directly from the cortex of lymphoid follicles in the bursa of Fabricius. Eur J Immunol. (1994) 24:458–63. 10.1002/eji.1830240229 8299695

[12] Schusser B Collarini EJ Yi H Izquierdo SM Fesler J Pedersen D. Immunoglobulin knockout chickens via efficient homologous recombination in primordial germ cells. Proc Natl Acad Sci USA. (2013) 110:20170–5. 10.1073/pnas.1317106110 24282302 PMC3864345

[13] Sayegh CE Ratcliffe MJ Perinatal deletion of B cells expressing surface Ig molecules that lack V(D)J-encoded determinants in the bursa of Fabricius is not due to intrafollicular competition. J Immunol. (2000) 164:5041–8. 10.4049/jimmunol.164.10.5041 10799859

[14] Sugiyama T Kohara H Noda M Nagasawa T. Maintenance of the hematopoietic stem cell pool by CXCL12-CXCR4 chemokine signaling in bone marrow stromal cell niches. Immunity. (2006) 25:977–88. 10.1016/j.immuni.2006.10.016 17174120

[15] Nagasawa T Hirota S Tachibana K Takakura N Nishikawa S Kitamura Y. Defects of B-cell lymphopoiesis and bone-marrow myelopoiesis in mice lacking the CXC chemokine PBSF/SDF-1. Nature. (1996) 382:635–8. 10.1038/382635a0 8757135

[16] Ma Q Jones D Borghesani PR Segal RA Nagasawa T Kishimoto T. Impaired B-lymphopoiesis, myelopoiesis, and derailed cerebellar neuron migration in CXCR4- and SDF-1-deficient mice. Proc Natl Acad Sci USA. (1998) 95:9448–53. 10.1073/pnas.95.16.9448 9689100 PMC21358

[17] Molyneaux KA Zinszner H Kunwar PS Schaible K Stebler J Sunshine MJ. The chemokine SDF1/CXCL12 and its receptor CXCR4 regulate mouse germ cell migration and survival. Development. (2003) 130:4279–86. 10.1242/dev.00640 12900445

[18] Liang TS Hartt JK Lu S Martins-Green M Gao JL Murphy PM. Cloning, mRNA distribution, and functional expression of an avian counterpart of the chemokine receptor/HIV coreceptor CXCR4. J Leukoc Biol. (2001) 69:297–305. 10.1189/jlb.69.2.297 11272281

[19] Read LR Cumberbatch JA Buhr MM Bendall AJ Sharif S. Cloning and characterization of chicken stromal cell derived factor-1. Dev Comp Immunol. (2005) 29:143–52. 10.1016/j.dci.2004.06.010 15450754

[20] Haertle S Alzuheir I Busalt F Waters V Kaiser P Kaufer BB. Identification of the receptor and cellular ortholog of the Marek's Disease Virus (MDV) CXC chemokine. Front Microbiol. (2017) 8:2543. 10.3389/fmicb.2017.02543 29326678 PMC5736565

[21] Nuthalapati NK Evans JD Taylor RL Branton SL Nanduri B Pharr GT. Transcriptomic analysis of early B-cell development in the chicken embryo. Poult Sci. (2019) 98:5342–54. 10.3382/ps/pez354 31237340 PMC6771548

[22] Becker M Hobeika E Jumaa H Reth M Maity PC. CXCR4 signaling and function require the expression of the IgD-class B-cell antigen receptor. Proc Natl Acad Sci USA. (2017) 114:5231–6. 10.1073/pnas.1621512114 28461496 PMC5441763

[23] Wilson SM Chambers AF. Experimental metastasis assays in the chick embryo. Curr Protoc Cell Biol. (2004) Chapter 19:Unit 19 16. 10.1002/0471143030.cb1906s21 18228449

[24] Ran FA Hsu PD Wright J Agarwala V Scott DA Zhang F. Genome engineering using the CRISPR-Cas9 system. Nat Protoc. (2013) 8:2281–308. 10.1038/nprot.2013.143 24157548 PMC3969860

[25] Wang H Qi M Cutler AJ. A simple method of preparing plant samples for PCR. Nucleic Acids Res. (1993) 21:4153–4. 10.1093/nar/21.17.4153 8371994 PMC310032

[26] Busalt F Untersuchungen zur Rolle von CXCR4 und CXCR5 in der B-Zell-Entwicklung beim Huhn (DVM doctoral thesis), Ludwig-Maximilians-University Munich, Germany (2013).

[27] Schusser B Yi H Collarini EJ Izquierdo SM Harriman WD Etches RJ. Harnessing gene conversion in chicken B cells to create a human antibody sequence repertoire. PLoS ONE. (2013) 8:e80108. 10.1371/journal.pone.0080108 24278246 PMC3837002

[28] Weill JC Reynaud CA. The chicken B cell compartment. Science. (1987) 238:1094–8. 10.1126/science.3317827 3317827

[29] Ratcliffe MJ. Antibodies, immunoglobulin genes and the bursa of Fabricius in chicken B cell development. Dev Comp Immunol. (2006) 30:101–18. 10.1016/j.dci.2005.06.018 16139886

[30] Sayegh CE Demaries SL Iacampo S Ratcliffe MJ. Development of B cells expressing surface immunoglobulin molecules that lack V(D)J-encoded determinants in the avian embryo bursa of fabricius. Proc Natl Acad Sci USA. (1999) 96:10806–11. 10.1073/pnas.96.19.10806 10485907 PMC17964

[31] Houssaint E Belo M Le Douarin NM. Investigations on cell lineage and tissue interactions in the developing bursa of Fabricius through interspecific chimeras. Dev Biol. (1976) 53:250–64. 10.1016/0012-1606(76)90227-X 992209

[32] Pink JR Vainio O Rijnbeek AM. Clones of B lymphocytes in individual follicles of the bursa of Fabricius. Eur J Immunol. (1985) 15:83–7. 10.1002/eji.1830150116 3871398

[33] Houssaint E Lassila O Vainio O. Bu-1 antigen expression as a marker for B cell precursors in chicken embryos. Eur J Immunol. (1989) 19:239–43. 10.1002/eji.1830190204 2649381

[34] Busillo JM Benovic JL. Regulation of CXCR4 signaling. Biochim Biophys Acta. (2007) 1768:952–63. 10.1016/j.bbamem.2006.11.002 17169327 PMC1952230

[35] Del Nagro CJ Otero DC Anzelon AN Omori SA Kolla RV Rickert RC. CD19 function in central and peripheral B-cell development. Immunol Res. (2005) 31:119–31. 10.1385/IR:31:2:119 15778510

[36] Lampisuo M Arstila TP Liippo J Lassila O. Expression of chL12 surface antigen is associated with cell survival in the avian bursa of Fabricius. Scand J Immunol. (1998) 47:223–8. 10.1046/j.1365-3083.1998.00291.x 9519860

