# Supplementary figures and images for: Blocking of the CXCR4-CXCL12 Interaction Inhibits the Migration of Chicken B Cells Into the Bursa of Fabricius
Source: Front Immunol. 2020 Jan 10;10:3057. doi: 10.3389/fimmu.2019.03057 (PMC6967738; doi:10.3389/fimmu.2019.03057)

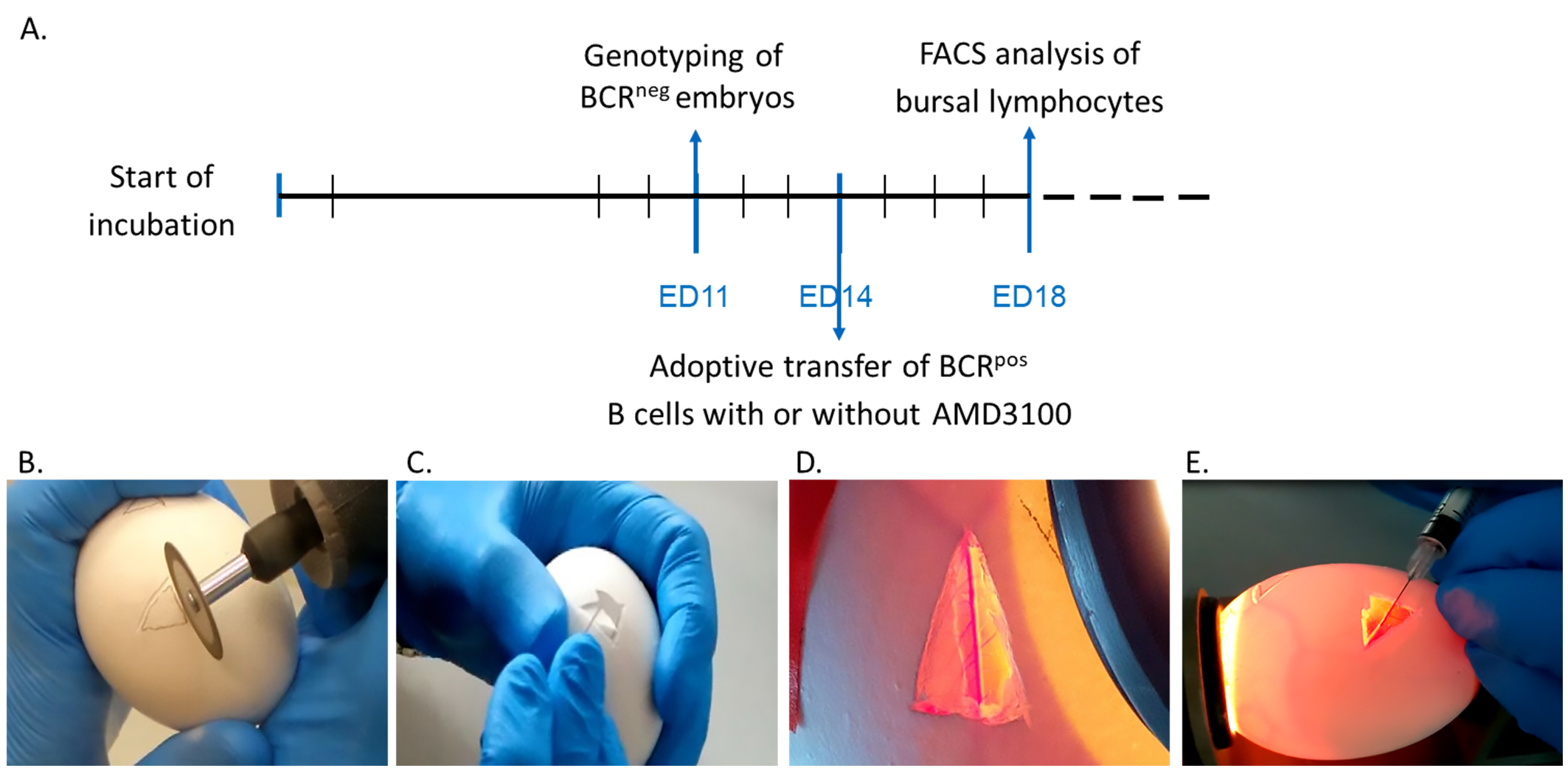

Supplement: Supplemental Figure 1 — In ovo blood sampling and adoptive cell transfer. (A) Eggs from a BCRpos/neg × BCRpos/neg breeding were set for incubation. On ED11 blood was taken in ovo for genomic DNA isolation and genotyping of the embryos in order to detect the BCRneg embryos. On ED14 splenocytes isolated from wt embryos were adoptively transferred into the BCRneg embryos. On ED18 the embryos were sacrificed and bursal lymphocyte populations were analyzed per flow cytometry. In ovo blood sampling but also adoptive cell transfer require drilling of a triangle over a stable vessel (B), dispatching of the triangle from the egg shell (C), viewing of the vessel after putting a drop of paraffin oil on the membrane (D), and approach of the vessel with a 30G needle (E). [file Image_1.JPEG]
