# Supplementary material for: Blocking of the CXCR4-CXCL12 Interaction Inhibits the Migration of Chicken B Cells Into the Bursa of Fabricius
Source: Front Immunol. 2020 Jan 10;10:3057. doi: 10.3389/fimmu.2019.03057 (PMC6967738; doi:10.3389/fimmu.2019.03057)
Supplement: Supplemental Table 1 — Primers used for the qRT-PCR for the analysis of the CXCR4 and CXCL12 expression in the embryonic bursa. [file Table_1.pdf]

Nr. of primer	Direction	Sequence	Amplification length (bp)
455 CXCR4	Forward	ctgtggctgacctcctctttg	86
456 CXCR4	Reverse	acacaggacatttccgaagtacc	86
457 CXCL12	Forward	ctcaagagcaacagcaagcaa	150
458 CXCL12	Reverse	gcccttaacgttctacccttga	150
322 18S	Forward	catgtctaagtacacacgggcggta	25
323 18S	Reverse	ggcgctgctggcatggtatta	20
